# Synthesis of chiral sulfoximine-based thioureas and their application in asymmetric organocatalysis

**DOI:** 10.3762/bjoc.8.164

**Published:** 2012-09-03

**Authors:** Marcus Frings, Isabelle Thomé, Carsten Bolm

**Affiliations:** 1 Institut für Organische Chemie, Landoltweg 1, RWTH Aachen University, 52074 Aachen, Germany

**Keywords:** anhydride opening, catalytic asymmetric Biginelli reaction, organocatalysis, sulfoximines, thioureas

## Abstract

For the first time, chiral sulfoximine derivatives have been applied as asymmetric organocatalysts. In combination with a thiourea-type backbone the sulfonimidoyl moiety leads to organocatalysts showing good reactivity in the catalytic desymmetrization of a cyclic *meso*-anhydride and moderate enantioselectivity in the catalytic asymmetric Biginelli reaction. Straightforward synthetic routes provide the newly designed thiourea-sulfoximine catalysts in high overall yields without affecting the stereohomogeneity of the sulfur-containing core fragment.

## Introduction

**Figure 1:**
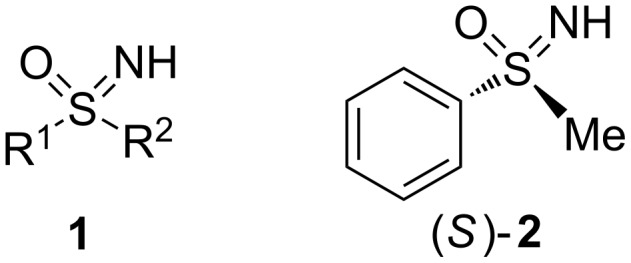
General structure of sulfoximines **1** and one of the enantiomers of *S*-methyl-*S*-phenylsulfoximine ((*S*)-**2**) used in this study.

Since their discovery in the middle of the last century, sulfoximines **1** (Figure 1) represent an important compound class among the comprehensive group of organosulfur reagents [1–4].

Being monoaza analogs of sulfones, sulfoximines have fascinated researchers from both academia and industry. Because of their interesting chemical properties and the biological activities found for several derivatives, the use of sulfoximines has been explored in numerous applications. For instance, in agricultural chemistry it was discovered that sulfoximines can improve plant growth or act as insecticides in crop protection [5–9]. Further exemplary contributions come from medicinal chemistry where sulfoximines show potential as enzyme inhibitors [10–14], and from materials science where they were evaluated as functional building blocks [15]. In addition, sulfoximines are most present in synthetic organic chemistry for various reasons and recent findings include their use as fluoromethylation reagents, as fluorophores or as directing groups [16–19]. Two quality characteristics make them particularly attractive for asymmetric synthesis: 1) The stereogenic sulfur atom which is stable towards many reaction conditions, and 2) the ease of functionalization at the adjacent nitrogen and carbon atoms which allows a great structural diversity of the sulfoximine motif. Hence, optically active compounds based on **2** have been utilized in the synthesis of pseudopeptides [20–24], and they have found widespread application in auxiliary-assisted diastereoselective transformations or as chiral ligands in enantioselective metal catalysis [25–33]. With respect to the latter field we have recently demonstrated that various ligands bearing a sulfonimidoyl moiety lead to excellent stereoselectivities in transition metal-catalyzed hydrogenations and carbon–carbon bond formations [34–36].

**Figure 2:**
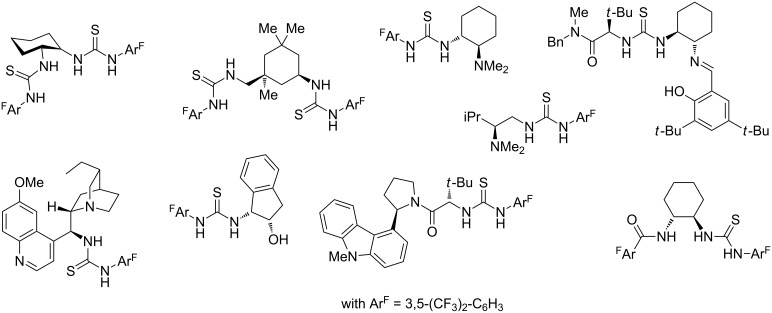
Structures of chiral mono- and bifunctional (bis-)thioureas that have been used as organocatalysts.

During the past decade, asymmetric organocatalysis had a tremendous impact on synthetic organic chemistry [37–41]. Yet, this field of research continues to grow, and the quest for new organic molecules which efficiently catalyze reactions in a highly enantioselective manner has no end in sight. In this context, thiourea-based organocatalysts have caught significant attention due to their ability to activate substrates through hydrogen-bonding [42–47]. Usually, these chiral thioureas are classified into several categories, for example, being mono- or bis-thioureas. Furthermore, they can be mono- or bifunctional with variably weak amine (primary, secondary, tertiary) or amide groups attached. Figure 2 illustrates a few selected examples of the aforementioned chiral thioureas which have successfully been applied in organic transformations with hydrogen bond accepting substrates.

Recently, we reported the enantioselective ring opening of cyclic *meso*-anhydrides and asymmetric Michael additions of 1,3-dicarbonyl compounds to nitroalkenes with thiourea-based organocatalysts [48–49]. Based on those studies and in the light of our long-standing interest in utilizing chiral sulfoximines in stereoselective catalytic reactions, we wondered about a molecular combination of the two successfully applied entities, thioureas and sulfoximines. To the best of our knowledge, such compounds have never been reported and thiourea(-like) catalysts with S-stereogenic sulfonimidoyl substituents are unknown. Herein, we present our first results concerning synthetic approaches towards such molecules and describe preliminary studies of two applications in asymmetric organocatalysis.

## Results and Discussion

**Scheme 1:**
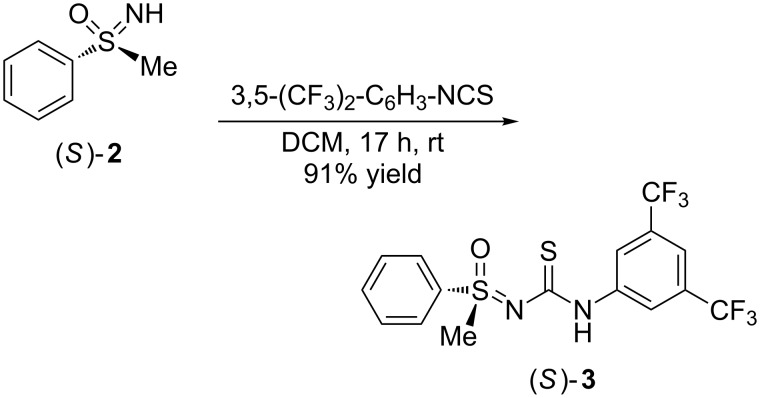
Synthesis of compound (*S*)-**3**.

Our investigations began with a very straightforward approach: Enantiopure (*S*)-*S*-methyl-*S*-phenylsulfoximine [(*S*)-**2**] was added to 3,5-bis(trifluoromethyl)phenyl isothiocyanate to provide product (*S*)-**3** (Scheme 1).

This kind of addition was first described by Wehr in 1965 who allowed a number of isothiocyanates to react with dimethylsulfoximine [50]. The chemistry of the resulting thiourea-like compounds, however, has remained rather unexplored until now. Only two patents from Dow Agrosciences report syntheses of related structures, their use as intermediate products, and their insecticidal activity [51–52]. Furthermore, only achiral or racemic sulfoximines have been applied, and the reactions were performed at elevated temperatures, such as 80 °C, or in a steam bath. The protocol introduced here is most simple: After addition of the isothiocyanate to a solution of sulfoximine (*S*)-**2** in dichloromomethane (DCM) the mixture was stirred at room temperature, and after a few hours, (*S*)-**3** started to precipitate. Removal of the solvent and washing of the product with *n*-pentane gave analytically pure (*S*)-**3** in 91% yield.

**Scheme 2:**
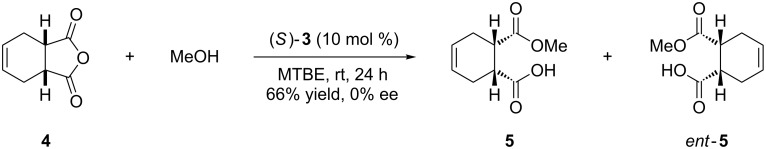
Organocatalytic desymmetrization of the cyclic anhydride **4** with (*S*)-**3**.

Although not capable of double hydrogen bonding [53], we wondered about the possible catalytic activity of (*S*)-**3**. Because it was known for chiral bifunctional amine-based sulfonamides that two hydrogen bond donors were not strictly required in the enantioselective organocatalytic ring opening of *meso*-anhydrides [48,54], this particular transformation was chosen as initial test reaction. Cyclic anhydride **4** served as starting material for a methanolysis in the presence of a catalytic amount of enantiopure (*S*)-**3** (Scheme 2). To our delight, the use of a combination of 10 equiv of methanol and 10 mol % of (*S*)-**3** in methyl *tert*-butyl ether (MTBE) at room temperature furnished the desired products, hemiesters **5** and *ent*-**5**, in good yield (66%) within 24 h. Disappointingly, however, the product was racemic.

In general, two concomitant events are discussed for bifunctional organocatalysts such as amino group-containing sulfonamides or thioureas: One is the activation of the anhydride carbonyl group by hydrogen bonding to the thiourea or sulfonamide unit, and the second relates to the activation of the alcohol by the basic nitrogen of the amine. Which effect dominates in the case of (*S*)-**3** – a carbonyl activation by a single hydrogen bond or an enhancement of the alcohol activity by the weakly basic sulfoximine nitrogen – remains to be elucidated. An organocatalytic activity of thiourea(-like) (*S*)-**3**, however, was clearly demonstrated.

**Scheme 3:**
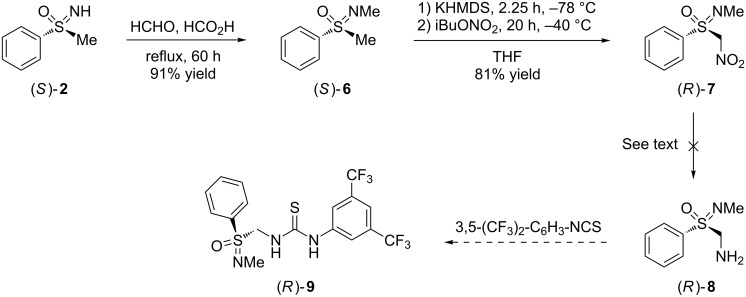
Attempted synthesis of sulfonimidoyl-substituted thiourea (*R*)-**9**.

Next, our attention was focused on sulfoximine-based thioureas with the potential of double hydrogen bond donation. Two structural alternatives were envisaged as represented by compounds (*R*)-**9** and (*S*)-**12**. In the first, the stereogenic sulfur of the sulfonimidoyl moiety was linked to the thiourea core by a methylene group, which originated from the methyl substituent of (*S*)-**2**. In the second, a linker connected the thiourea backbone with the sulfonimidoyl nitrogen. Preference was given to the first strategy, because in structures such as (*R*)-**9** the stereogenic center was rather close to the thiourea hydrogen bridge binding site. Scheme 3 summarizes our intended preparative approach towards (*R*)-**9** via *N*-methyl-α-aminosulfoximine (*R*)-**8**, which unfortunately, remained unsuccessful.

Heating of (*S*)-**2** with 2 equiv of formaldehyde in formic acid under Eschweiler–Clarke conditions led to *N*-methylated sulfoximine (*S*)-**6** in 91% yield [55–56]. The α-nitro group was introduced under conditions reported by Wade [57]. Thus, deprotonation of (*S*)-**6** in tetrahydrofuran (THF) with potassium bis(trimethylsilyl)amide (KHMDS) at −78 °C and trapping the resulting carbanion with isobutyl nitrate at −40 °C afforded nitrosulfoximine (*R*)-**7** in 81% yield. The product proved rather stable at low temperature and could be stored in the fridge without decomposition for several months. All attempts, however, to reduce the α-nitro group of (*R*)-**7** to the corresponding amino substituent (as in (*R*)-**8**) failed [58]. None of the reductive conditions, which included (1) hydrogenation over palladium/charcoal, (2) applying samarium diiodide with methanol as proton source, and (3) using a combination of zinc powder with hydrochloric acid or calcium chloride in aqueous ethanol allowed the isolation of α-aminosulfoximine (*R*)-**8**. In all cases the starting material was fully consumed and degraded. As decomposition products *N*-methylbenzenesulfinamide and diphenyl disulfide were identified and isolated. Hence, α-aminosulfoximine (*R*)-**8** could not be obtained, and the synthesis of the sulfoximine-based thiourea (*R*)-**9** had to remain uncompleted.

**Scheme 4:**
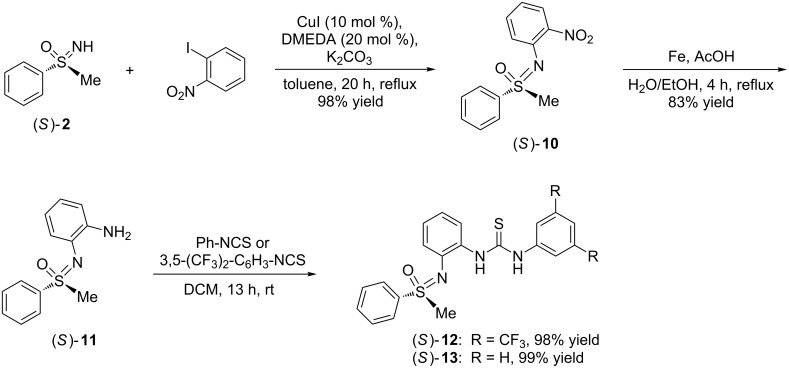
Synthesis of the sulfonimidoyl-containing thioureas (*S*)-**12** and (*S*)-**13**.

Next, the second strategy was approached, and thiourea/sulfoximine derivatives (*S*)-**9** and (*S*)-**13** became synthetic targets (Scheme 4). In both the stereogenic fragments are linked to the thiourea core via the sulfonimidoyl nitrogen. They differ in their aryl substitution pattern.

Copper-catalyzed arylation of (*S*)-**2** with 2-iodonitrobenzene gave rise to coupling product (*S*)-**10** in excellent yield (98%). After subsequent reduction of the aromatic nitro group to give aniline (*S*)-**11** in 83% yield [35,59–60], two isothiocyanates, namely phenyl isothiocyanate and 3,5-bis(trifluoromethyl)phenyl isothiocyanate, were added. Both thiourea formations went very well, and the desired products (*S*)-**12** and (*S*)-**13** were obtained in almost quantitative yields (98% and 99%, respectively).

**Scheme 5:**
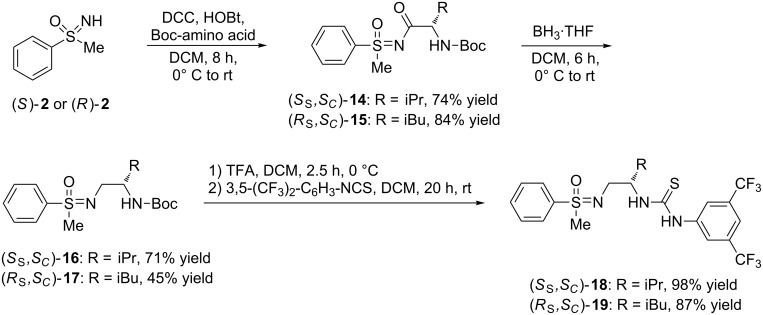
Syntheses of ethylene-linked sulfonimidoyl-containing thioureas (*S*_S_,*S*_C_)-**18** and (*R*_S_,*S*_C_)-**19**.

In (*S*)-**12** and (*S*)-**13** the 1,2-benzene linker connects both functional groups – the stereogenic sulfonimidoyl group and the thiourea core – in a rather rigid manner. In order to allow a higher degree of conformational flexibility and with the goal to determine the effects of structural rigidity and the presence of additional stereogenic centers, alternative molecules with substituted ethylene linkers ((*S*_S_,*S*_C_)-**18** and (*R*_S_,*S*_C_)-**19**) were designed. Their syntheses are outlined in Scheme 5, which also underlines the value of the highly modular preparative approach towards such compounds leading to a variety of molecules by using closely related synthetic protocols.

In the syntheses of (*S*_S_,*S*_C_)-**18** and (*R*_S_,*S*_C_)-**19** we benefited from our expertise in preparing sulfoximine-based pseudopeptides [20–21]. Hence, the reaction of (*S*)-**2** with *N*-Boc protected L-valine, *N*,*N*'-dicyclohexylcarbodiimide (DCC) and hydroxybenzotriazole (HOBt) provided (homochiral) (*S*_S_,*S*_C_)-**14** in good yield (74%). Already at this early stage we wondered, which of the two stereogenic centers in the resulting thioureas – the one at the sulfur atom or the one stemming from the amino acid – would determine the absolute configuration of the catalysis products. For this reason, a heterochiral product was prepared by coupling (*R*)-**2** (the mirror image of the previously applied sulfoximine) with *N*-Boc protected L-leucine to give (*R*_S_,*S*_C_)-**15** (84% yield). Reduction of the two *N*-acylated sulfoxmines (*S*_S_,*S*_C_)-**14** and (*R*_S_,*S*_C_)-**15** with borane-THF complex led to removal of the carbonyl groups and established the desired substituted ethylene bridges in (*S*_S_,*S*_C_)-**16** and (*R*_S_,*S*_C_)-**17** (71% and 45% yield, respectively). Subsequently, the *N*-Boc groups were smoothly cleaved upon treatment with trifluoroacetic acid (TFA), and for both substrates full conversion was observed after 2.5 h. An aqueous work-up followed by the addition of 3,5-bis(trifluoromethyl)phenyl isothiocyanate to each crude primary amine completed the syntheses of ethylene-linked sulfonimidoyl-containing thioureas (*S*_S_,*S*_C_)-**18** and (*R*_S_,*S*_C_)-**19** providing them in very good yields (98% and 87%, respectively) over two steps.

One representative of each class was briefly tested in the desymmetrization of anhydride **4**. Under the conditions described above for organocatalyst (*S*)-**3** two catalyses were performed with (*S*)-**12** (10 mol %) and (*R*_S_,*S*_C_)-**19** (5 mol %). Whereas benzene-bridged sulfonimidoyl-containing thiourea (*S*)-**12** provided the product (**5**/*ent*-**5**) in a comparable yield (67%) as (*S*)-**3**, the reactivity of (*R*_S_,*S*_C_)-**19** was remarkably higher. Even though in the reaction with (*R*_S_,*S*_C_)-**19** the catalyst loading was lower, the product was isolated in a better yield (81%) under identical conditions. Unfortunately, both catalyses led to racemic hemiesters (**5**/*ent*-**5**). Apparently, both thioureas proved inappropriate for this reaction, and no further efforts were made to improve the anhydride desymmetrization with these types of organocatalysts.

Next, the catalysis screening was focused on Biginelli reactions, which provide access to dihydropyrimidines by three-component condensations of aldehydes, urea-type substrates and enolisable carbonyl compounds. Because of the pharmacological relevance of the products, considerable research has been directed towards asymmetric approaches of these inherently chiral heterocycles [61–63]. Until recently, enantioenriched dihydropyrimidines could only be obtained by special resolution methods which were not generally applicable. The search for a truly catalytic asymmetric Biginelli reaction with high enantiocontrol has proven very challenging for a long time and useful protocols have just recently been developed. They involve both metal catalysis [64–65] as well as organocatalysis. In the latter field, chiral phosphoric acids [66–67], bicyclic diamines [68], bifunctional thioureas [69], proline ester salts [70], pyrrolidinyl tetrazoles [71], and a quinine-derived amine [72] were reported to catalyze the condensation reaction in an asymmetric fashion.

Using a slightly modified procedure of the thiourea-catalyzed Biginelli reaction developed by Miao and Chen [69], we chose the condensation between urea, benzaldehyde (1.5 equiv), and ethyl acetoacetate (3 equiv) to give dihydropyrimidine **20** (Table 1) as test reaction. As catalysts, substoichiometric quantities of the sulfonimidoyl-containing thioureas in combination with 10 mol % of trifluoroacetic acid (TFA) were applied. The results are summarized in Table 1.

**Table 1:** Evaluation of the sulfonimidoyl-containing thiourea organocatalysts in the asymmetric Biginelli reaction to afford scalemic dihydropyrimidinone (*S*)-**20**.

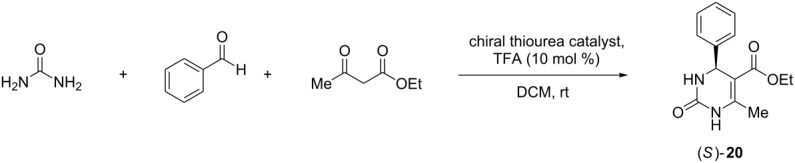						

entry	chiral thiourea	catalyst loading (mol %)/ concentration (mol/L)	time (d)	substrate concentration (mol/L)^a^	yield (%)	er (%)^b^

1	(*S*)-**3**	10/0.025	3	0.25	28	50:50
2	(*S*_S_,*S*_C_)-**18**	10/0.025	3	0.25	32	50:50
3	(*R*_S_,*S*_C_)-**19**	10/0.025	3	0.25	30	50:50
4	(*S*)-**12**	10/0.025	3	0.25	42	58:42
5	(*S*)-**13**	10/0.025	3	0.25	21	57:43
6	(*S*)-**12**	10/0.025	5	0.25	89	58:42
7	(*S*)-**13**	10/0.025	5	0.25	40	57:43
8	(*S*)-**12**	20/0.05	5	0.25	90	59:41
9	(*S*)-**13**	20/0.05	5	0.25	42	57:43
10	(*S*)-**12**	10/0.0025	5	0.025	92	72:28
11	(*S*)-**12**	1/0.0025	5	0.25	53	51:49

^a^With respect to urea. ^b^Determined by HPLC analysis with a chiral stationary phase.

The experiment with 10 mol % of chiral organocatalyst (*S*)-**3** served as starting point (Table 1, entry 1). The low product yield (28%) even after a long reaction time (3 days) revealed the insufficient catalytic activity of this thiourea derivative. Furthermore, product **20** was racemic. Analogous results were observed with the two ethylene-bridged sulfonimidoyl-containing thioureas (*S*_S_,*S*_C_)-**18** and (*R*_S_,*S*_C_)-**19** when the catalyses were conducted under identical conditions (Table 1, entries 2 and 3). Racemic dihydropyrimidinone **20** was obtained in 32% and 30% yields, respectively. Next, we focused on the application of thioureas (*S*)-**12** and (*S*)-**13** having a more rigid benzene linker (Table 1, entries 4 and 5). Although the yields were only moderate (42% and 21%, respectively), we delightfully noted that product **20** was enantioenriched for the first time (with the *S*-enantiomer being formed in preference). With enantiomer ratios of 58:42 and 57:43 the enantioselectivities were still low, but we envisaged that improvements were possible by catalyst structure optimizations and reaction condition adjustments. Apparently, the substituents at the 3,5-positions of the phenyl rings at the thiourea moieties (CF_3_ versus H) had only a marginal effect on the stereoselectivity of the Biginelli reaction. However, they affected the catalyst activity as indicated by the doubled yield in the catalysis with (*S*)-**12** compared to the one with (*S*)-**13** (42% versus 21%). This increase in yield might have been because of a higher acidity of (*S*)-**12** due to the presence of the two CF_3_ substituents on the thiourea aryl [73]. Prolonging the reaction time from 3 to 5 days raised the yields (Table 1, entries 6 and 7), and now, for example, the catalysis with (*S*)-**12** gave the product in 89% yield (Table 1, entry 6). The enantiomer ratios remained unaffected. Increasing the catalyst loading from 10 to 20 mol % had essentially no effect on both yield and enantioselectivity (Table 1, entries 8 and 9).

In previous studies it was found that the enantioselectivity in some thiourea-catalyzed reactions was dependent on the concentration of the substrates and that in several cases the enantiomeric excess could significantly be improved by dilution [48,74–76]. This effect was attributed to the formation of less active aggregates by thioureas self-association at high concentrations resulting in lower enantioselectivities [77]. Interestingly, a dilution effect was also observed in our study. Thus, changing the molar substrate (urea) concentration from 0.25 mol/L to 0.025 mol/L caused a distinct improvement of the enantiomeric excess and the enantiomer ratio of the product **20** raised from 58:42 to 72:28 (Table 1, entry 6 vs entry 10). In addition, product **20** was isolated in a slightly better yield (92%). The attempt to reduce the catalyst loading from 10 mol % to 1 mol % (while retaining its concentration at 0.0025 mol/L; Table 1, entry 10 vs entry 11) remained unsuccessful resulting in 53% of **20** with an er of only 51:49. Probably, the reaction path was affected by the 10-fold higher substrate concentration, which led to this unsatisfying enantioselectivity.

These results allow drawing a few preliminary conclusions related to the structural requirements of the chiral sulfonimidoyl-containing thioureas for achieving enantiocontrol in subsequent asymmetric catalysis studies. For example, it is noteworthy that compared to most other organocatalysts of the thiourea-type, which commonly have the stereogenic center directly attached to one of the NH groups of the thiourea moiety (Figure 2), the nearest stereogenic center in (*S*)-**12**, (*S*)-**13**, (*S*_S_,*S*_C_)-**18** and (*R*_S_,*S*_C_)-**19** is relatively remote from the thiourea core, which most likely serves as binding site for the substrate. Nevertheless, a remarkable enantiomeric ratio of 78:22 has already been achieved in the Biginelli reaction, which is known to be difficult to control. Thus, bringing the sulfonimidoyl group into closer proximity to the thiourea core might be beneficial for achieving a higher enantiocontrol, but it does not appear to be essential. Another point should be emphasized here as well. Compounds such as (*S*)-**12** and (*S*)-**13** are some of the very rare examples of chiral organocatalysts, whose only stereocontrolling element is not an asymmetrically substituted carbon but rather a stereogenic sulfur center [78–80].

With respect to the properties of the linker between the sulfonimidoyl group bearing the stereogenic sulfur atom and the thiourea unit (aryl vs substituted ethylene as is **12** and **13** vs **18** and **19**) it became clear that conformational rigidity is beneficially leading to higher enantioselectivities. Perhaps, substituted arene backbones could be used for inducing enhanced positive effects on both units through electronic fine-tuning.

The additional stereogenic centers in the ethylene linker as in (*S*_S_,*S*_C_)-**18** and (*R*_S_,*S*_C_)-**19** had none or at best only a minor impact on the stereochemistry-determining path despite the fact that they were relatively close to the thiourea substrate binding site. Thus, reducing molecular flexibility by incorporation of plain arenes as linkers appears to be more important than conformational fixation through additional stereogenic centers in the catalyst backbone.

Overall, promising results have been achieved, which shall be taken as stimulus for further investigations of structurally related sulfonimidoyl-containing thioureas. In that catalyst design, the aforementioned aspects related to structure, activity and stereoselectivity must then be considered and serve as guideline.

## Conclusion

In summary, we introduced enantiopure sulfoximines to the field of organocatalysis. The present work demonstrates that the addition of phenyl isothiocyanates to suitable amino-functionalized sulfoximines or to the sulfoximine nitrogen itself proceeds in high yields to give sulfonimidoyl-containing thiourea-type structures. While these compounds show good catalytic activity but no enantiocontrol in the ring opening of a cyclic *meso*-anhydride, low to moderate enantioselectivities have been achieved in the catalytic asymmetric Biginelli reaction leading to a dihydropyrimidinone. In the future we aim to expand the scope of the chiral sulfonimidoyl-containing thiourea framework and hope to find more applications of these interesting molecules in asymmetric organocatalysis.

## Supporting Information

File 1 Experimental section and full characterization data of all new compounds.
