# Supplementary material for: Synthesis of chiral sulfoximine-based thioureas and their application in asymmetric organocatalysis
Source: Beilstein J Org Chem. 2012 Sep 3;8:1443–51. doi: 10.3762/bjoc.8.164 (PMC3458769; doi:10.3762/bjoc.8.164)
Supplement: File 1 — Experimental section and full characterization data of all new compounds. [file Beilstein_J_Org_Chem-08-1443-s001.pdf]

Supporting Information

for

Synthesis of chiral sulfoximine-based thioureas and their application in asymmetric organocatalysis

Marcus Frings, Isabelle Thomé and Carsten Bolm*

Address: Institut für Organische Chemie, Landoltweg 1, RWTH Aachen University, 52074 Aachen, Germany

Email: Carsten Bolm* - carsten.bolm@oc.rwth-aachen.de

* Corresponding author

Experimental section and full characterization data of all new compounds

General information: Flash chromatography was carried out with Merck silica gel 60 (63–100 mesh). Analytical thin layer chromatography was performed with aluminium sheets silica gel 60 F_{254} (Merck), and the products were visualized by a basic aqueous solution of potassium permanganate. Optical rotation measurements were conducted with a Perkin-Elmer model 241 polarimeter (room temperature, λ = 589 nm) and are given in $deg \cdot cm^3 \cdot g^{-1} \cdot dm^{-1}$; concentration *c* is listed in $g \cdot (100\ mL)^{-1}$. Nuclear magnetic resonance (NMR) spectra were recorded on a Varian VNMRS 400 (^{1}H NMR: 400 MHz, ^{13}C NMR: 100 MHz, ^{19}F NMR: 376 MHz) spectrometer. Chemical shifts (δ) are given in ppm relative to tetramethylsilane (δ = 0 ppm) or to the solvent residual peak of deuterated chloroform (^{1}H NMR: δ = 7.26 ppm, ^{13}C NMR: δ = 77.0 ppm) or dimethyl sulfoxide (^{1}H NMR: δ = 2.50 ppm, ^{13}C NMR: δ = 39.5 ppm) as internal standard or trichlorofluoromethane (^{19}F NMR: δ = 0 ppm) as external standard. Coupling constants *J* are reported in Hz, and coupling patterns are described as br = broad, s = singlet, d = doublet, t = triplet, q = quartet, m = multiplet. Infrared (IR) spectra were recorded with attenuated total reflectance technique (ATR) on a Spectrum 100 spectrometer with an attached UATR device Diamond KRS-5, and wave numbers ν of the absorptions are reported in cm^{-1} with indicated relative intensities: w (weak, 0–33%), m (medium, 34–66%) and s (strong, 67–100%). Regular mass (MS) spectra were acquired on a Finnigan SSQ 7000 spectrometer [electron ionization (EI), 70 eV; chemical ionization (CI), 100 eV], high resolution mass (HRMS) spectra were recorded on a Finnigan MAT 95 spectrometer (EI) and a Thermo Scientific LTQ Orbitrap XL [electrospray ionization (ESI)], and peaks are listed according to their *m*/*z* values with their intensities given in percentage. Elemental analyses (EA) were measured on an Elementar Vario EL instrument. Melting points (mp) were determined in open-end capillary tubes on a Büchi B-540 melting point apparatus. Analytical high-performance liquid chromatography (HPLC) measurements for the determination of enantiomers were performed with an Agilent 1200-series system and a chiral stationary phase (Chiralcel OD-H: 250 mm × 4.6 mm) from Chiral Technologies Inc. Gas chromatographic (GC) analyses for the determination of enantiomers were performed on a Hewlett Packard 5890 Series II device and a HP 3396 integrator using a chiral stationary phase (Lipodex E: 50 m × 0.25 mm).

Both enantiomers of *S*-methyl-*S*-phenylsulfoximine (**2**) [1], (*S*)-*N*,*S*-dimethyl-*S*-phenylsulfoximine [(*S*)-**6**] [2], (*R*)-*N*-methyl-*S*-nitromethyl-*S*-phenylsulfoximine [(*R*)-**7**] [3], (*S*)-*N*-(2-nitrophenyl)-*S*-methyl-*S*-phenylsulfoximine [(*S*)-**10**] [4], (*S*)-*N*-(2-aminophenyl)-*S*-methyl-*S*-phenylsulfoximine [(*S*)-**11**] [4], and (S_S,S_C)-*N*-{2-[(*tert*-butyloxycarbonyl)amino]-3-methylbutyl}-*S*-methyl-*S*-phenylsulfoximine [(S_S,S_C)-**16**] [5] were prepared according to

literature protocols, and all spectroscopic data matched those reported. 4-Cyclohexene-*cis*-1,2-dicarboxylic acid anhydride (**4**) was kindly donated by Dr. Ingo Schiffers. Benzaldehyde and trifluoroacetic acid were purified by distillation. Dichloromethane was distilled from calcium hydride in a nitrogen atmosphere. All other reagents were purchased from commercial suppliers und used without further purification. The supplier's specified purities of the reagents were calculated into the reaction batches.

(*S*)-*N*-[3,5-Bis(trifluoromethyl)phenyl]-*N*'-[methyl(oxido)phenyl-λ^4-sulfanylidene] thiourea [(*S*)-3]:

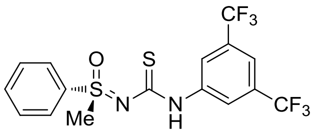

To a solution of (*S*)-*S*-methyl-*S*-phenylsulfoximine [(*S*)-**2**, 155.1 mg, 0.9992 mmol] in dry dichloromethane (2 mL) was dropwise syringed 3,5-bis(trifluoromethyl)phenyl isothiocyanate (226 μL, 1.20 mmol). Then, after some time, the precipitation of the product started. The reaction mixture was stirred for a total of 17 h. The solvent was evaporated under reduced pressure, and the obtained residue was washed with *n*-pentane to give the analytically pure product as white solid.

Yield 91% (385.8 mg, 0.9048 mmol); mp 187–188 °C; $[\alpha]_D$ −38.0 (*c* 1.1, $CHCl_3$); ^{1}H NMR (400 MHz, $CDCl_3$) 3.55 (s, 3H), 7.56–7.65 (m, 3H), 7.67–7.74 (m, 1H), 7.92–8.04 (m, 4H), 8.80 (s br, 1H); ^{13}C NMR (100 MHz, $CDCl_3$) 44.5, 117.4, 121.4, 123.0 (q, 272.6 Hz), 127.3, 129.8, 132.0 (q, 33.5 Hz), 134.0, 137.7, 139.9, 186.2; ^{19}F NMR (376 MHz, $CDCl_3$) −62.97 (s, 6F); IR 3138 (w), 1618 (w), 1558 (m), 1539 (m), 1469 (m), 1450 (m), 1410 (w), 1363 (s), 1278 (s), 1217 (s), 1163 (s), 1122 (s), 1093 (s), 1039 (s), 966 (m), 887 (m), 746 (s), 732 (s), 698 (m), 680 (s); MS (EI) 426 (10) $[M]^+$, 411 (100), 348 (7), 317 (34), 301 (15), 255 (27), 198 (8), 157 (21), 140 (33), 125 (47), 109 (28), 77 (17); EA calcd. (for $C_{16}H_{12}F_6N_2OS_2$) C 45.07, H 2.84, N 6.57, found C 44.96, H 2.67, N 6.59.

(*S*)-*N*-[3,5-Bis(trifluoromethyl)phenyl]-*N*'-[2'-(*S*-methyl-*S*-phenylsulfonimidoyl)phenyl]-thiourea [(*S*)-12]:

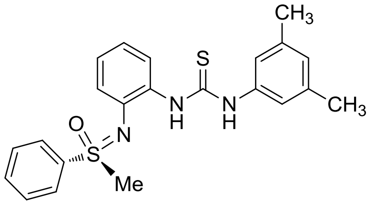

To a solution of the sulfoximine (*S*)-**11** (0.145 g, 0.589 mmol) in dry dichloromethane (2.5 mL) was dropwise syringed 3,5-bis(trifluoromethyl)phenyl isothiocyanate (144 μL, 0.765 mmol) at 0 °C. The solution was stirred at rt for 13 h. After evaporation of the solvent under reduced pressure the obtained residue was processed by column chromatography (*n*-pentane/ethyl acetate 3/1). The pale yellow product was dissolved in a small amount of dichloromethane and then precipitated as white solid by addition of *n*-hexane.

Yield 98% (299.4 mg, 0.5785 mmol); mp 94–96 °C; $[\alpha]_D$ −40.0 (*c* 1.6, $CHCl_3$); ^{1}H NMR (400 MHz, $CDCl_3$) 3.26 (s, 3H), 6.97 (td, 7.7 Hz, 1.1 Hz, 1H), 7.06 (td, 7.8 Hz, 1.2 Hz, 1H), 7.18 (d, 7.5 Hz, 1H), 7.45 (d, 7.2 Hz, 1H), 7.53 (t, 7.6 Hz, 2H), 7.59–7.66 (m, 2H), 7.92–7.99 (m, 4H), 8.57 (s br, 1H), 8.64 (s br, 1H); ^{13}C NMR (100 MHz, $CDCl_3$) 45.5, 118.7, 122.8, 122.9 (q, 272.5 Hz), 123.6, 124.0, 124.9, 127.9, 128.1, 129.8, 130.3, 131.8 (q, 33.6 Hz), 133.8, 138.3, 138.8, 140.2, 179.7; ^{19}F NMR (376 MHz, $CDCl_3$) −62.96 (s, 6F); IR 3271 (w), 1526 (s), 1474 (m), 1447 (m), 1380 (s), 1274 (s), 1170 (s), 1126 (s), 1048 (w), 1019 (m), 978 (m), 886 (m), 743 (s), 681 (s); MS (EI) 517 (55) $[M]^+$, 377 (93), 362 (31), 344 (60), 271 (96), 246 (72), 213 (28), 156 (47), 141 (100), 125 (38), 106 (41); EA calcd. (for $C_{22}H_{17}F_6N_3OS_2$) C 51.06, H 3.31, N 8.12, found C 51.18, H 3.37, N 8.00.

(*S*)-*N*-[2-(*S*-Methyl-*S*-phenylsulfonimidoyl)phenyl]-*N*’-phenylthiourea [(*S*)-13]:

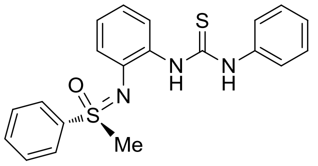

To a solution of the sulfoximine (*S*)-**11** (0.145 g, 0.589 mmol) in dry dichloromethane (2.5 mL) was dropwise syringed phenyl isothiocyanate (82.9 μL, 0.766 mmol) at 0 °C. The solution was stirred at rt for 13 h. After evaporation of the solvent under reduced pressure the obtained residue was processed by column chromatography (*n*-pentane/ethyl acetate 3/1), and the product was obtained as beige solid.

Yield 99% (222.5 mg, 0.5831 mmol); mp 79–82 °C; $[\alpha]_D$ −121.4 (*c* 0.7, $CHCl_3$); ^{1}H NMR (300 MHz, $CDCl_3$) 2.91 (s, 3H), 6.64–6.90 (m, 3H), 7.07–7.24 (m, 1H), 7.25–7.46 (m, 6H), 7.42–7.56 (m, 1H), 7.70 (d, J = 7.6 Hz, 2H), 8.34 (s, 2H), 8.91 (s, 1H); ^{13}C NMR (75 MHz, $CDCl_3$) 45.9, 121.1, 121.7, 121.9, 125.3, 125.6, 126.9, 128.2, 129.6, 129.7, 131.6, 133.6, 136.3, 137.2, 138.2, 178.0; IR 3258 (m), 2322 (m), 2099 (s), 1705 (s), 1589 (s), 1524 (w), 1447 (w), 1364 (m), 1249 (w), 1096 (m), 1021 (w), 957 (m), 911 (w), 736 (s); MS (EI) 382 (4) $[M]^+$, 288 (10), 246 (100), 255 (16), 208 (19), 141 (44), 135 (90), 106 (43), 77 (62); HRMS (ESI) calcd. (for $C_{20}H_{20}N_3OS_2$) 382.1042, found 382.1037.

(R_S,S_C)-*N*-{2-[(*tert*-Butyloxycarbonyl)amino]-4-methyl-1-oxopentyl}-*S*-methyl-*S*-phenylsulfoximine [(R_S,S_C)-15]:

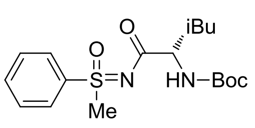

To a solution of (*R*)-*S*-methyl-*S*-phenylsulfoximine [(*R*)-**2**, 0.518 g, 3.34 mmol], hydroxybenzotriazole (0.460 g, 3.34 mmol), and *N*-Boc protected L-leucine (0.772 g, 3.34 mmol) in dichloromethane (20 mL) was added a solution of *N*,*N*'-dicyclohexylcarbodiimide in dichloromethane (3.4 mL, 3.4 mmol, *c* 1.0 mol/L). After 1 h the reaction mixture was warmed to rt and stirred for

additional 7 h. The precipitate was filtered off, washed with dichloromethane and discarded. The solvent of the combined solutions was removed under reduced pressure, and the product was purified by column chromatography (*n*-pentane/ethyl acetate 1/1) to give the white solid.

Yield 84% (1.034 g, 2.806 mmol); mp 110–112 °C; $[\alpha]_D$ −1.4 (*c* 1.6, $CHCl_3$); ^1H NMR (400 MHz, $CDCl_3$) 0.93 (d, 6.5 Hz, 3H), 0.95 (d, 6.3 Hz, 3H), 1.44 (s, 9H), 1.45–1.52 (m, 1H), 1.64–1.81 (m, 2H), 3.36 (s, 3H), 4.27–4.37 (m, 1H), 5.04 (d, 8.5 Hz, 1H), 7.57–7.63 (m, 2H), 7.65–7.71 (m, 1H), 7.99 (d, 7.6 Hz, 2H); ^{13}C NMR (100 MHz, $CDCl_3$) 22.1, 23.2, 25.0, 28.5, 42.6, 44.3, 55.5, 79.2, 127.1, 129.6, 133.8, 138.4, 155.6, 181.9; IR 3382 (m), 2976 (m), 2932 (m), 1682 (s), 1618 (s), 1526 (s), 1447 (m), 1414 (w), 1391 (w), 1366 (m), 1300 (s), 1257 (s), 1215 (s), 1166 (s), 1100 (s), 1038 (s), 1014 (s), 992 (s), 965 (s), 854 (s), 833 (s), 778 (m), 758 (s), 735 (s), 685 (s); MS (EI) 369 (1) $[M + H]^+$, 326 (8), 295 (4), 186 (10), 182 (100), 141 (28), 140 (18), 125 (7), 77 (4), 57 (26); MS (CI) 369 (100) $[M + H]^+$; HRMS (EI) calcd. (for $C_{18}H_{29}N_2O_4S$) 369.1843, found 369.1842.

(R_S,S_C)-*N*-{2-[(*tert*-Butyloxycarbonyl)amino]-4-methylpentyl}-*S*-methyl-*S*-phenylsulfox-imine [(R_S,S_C)-17]:

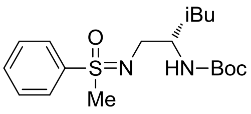

To a solution of the sulfoximine [(R_S,S_C)-**15**] (500.0 mg, 1.357 mmol) in dry dichloromethane (13 mL) was slowly added a borane-tetrahydrofuran complex (4.1 mL, 4.1 mmol, *c* 1.0 mol/L) at 0 °C. The reaction mixture was stirred at this temperature for 3 h, at rt for additional 3 h, and then carefully quenched with water. The aqueous phase was extracted three times with dichloromethane, and the combined organic phases were dried over magnesium sulfate. After evaporation of the solvent under reduced pressure the residue was processed by column chromatography (*n*-pentane/ethyl acetate 1/1). A second column chromatography (diethyl ether) yielded the product as colourless oil.

Yield 45% (214.6 mg, 0.6053 mmol); $[\alpha]_D$ −77.9 (*c* 2.2, $CHCl_3$); ^1H NMR (400 MHz, $CDCl_3$) 0.88 (d, 6.9 Hz, 3H), 0.90 (d, 6.7 Hz, 3H), 1.31–1.51 (m, 11H), 1.56–1.68 (m, 1H), 2.90–2.98 (m, 2H), 3.07 (s, 3H), 3.65–3.80 (m, 1H), 4.85 (d, 7.8 Hz, 1H), 7.51–7.63 (m, 3H), 7.86–7.91 (m, 2H); ^{13}C NMR (100 MHz, $CDCl_3$) 22.7, 23.0, 24.9, 28.6, 42.1, 45.0, 47.3, 49.5, 78.7, 128.5, 129.3, 132.8, 139.6, 155.5; IR 3363 (m), 3009 (m), 2957 (s), 2928 (s), 2869 (m), 1703 (s), 1501 (s), 1448 (m), 1387 (m), 1366 (m), 1242 (s), 1169 (s), 1085 (m), 1070 (m), 984 (m), 752 (s), 690 (m); MS (EI) 355 (3) $[M + H]^+$, 281 (8), 168 (100), 141 (71), 140 (24), 57 (24); MS (CI) 355 (100) $[M + H]^+$; HRMS (EI) calcd. (for $C_{18}H_{31}N_2O_3S$) 355.2050, found 355.2048.

(S_S,S_C)-*N*-[3,5-Bis(trifluoromethyl)phenyl]-*N*'-[1'-(*S*-methyl-*S*-phenylsulfonimidoyl-methyl)-2'-methylpropyl]thiourea [(S_S,S_C)-18]:

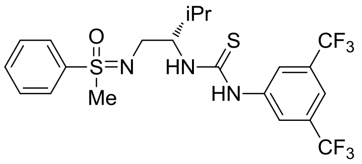

The sulfoximine (S_S,S_C)-**16** (93.5 mg, 0.275 mmol) was dissolved in dry dichloromethane (3 mL), slowly treated with trifluoroacetic acid (0.6 mL, 8 mmol) at 0 °C, and stirred at this temperature for 2.5 h. After evaporation of all volatiles under reduced pressure the oily residue was taken up in dichloromethane and aqueous sodium hydroxide (*c* 0.75 mol/L). The aqueous phase was extracted three times with dichloromethane, and the combined organic phases were dried over magnesium sulfate. The solvent was evaporated under reduced pressure, and the pale yellow, oily deprotected intermediate was dried in high vacuum for 1 h. It was then dissolved in dry dichloromethane (2 mL), and 3,5-bis(trifluoromethyl)phenyl isothiocyanate (51 μL, 0.27 mmol) was dropwise syringed to this solution at 0 °C. After stirring at rt for 23 h the solvent was removed, and the product was purified by column chromatography (*n*-pentane/ethyl acetate 2/1 to 1/1 with 1% triethylamine) to give the beige solid.

Yield 98% (137.6 mg, 0.2690 mmol) over two steps; mp 101–102 °C; $[\alpha]_D$ 6.1 (*c* 0.5, $CHCl_3$); ^1H NMR [400 MHz, $(CD_3)_2SO$] 0.88 (d, 6.8 Hz, 3H), 0.91 (d, 6.8 Hz, 3H), 2.10–2.25 (m, 1H), 2.90 (dd, 12.4 Hz, 4.0 Hz, 1H), 2.98 (dd, 12.3 Hz, 5.6 Hz, 1H), 3.18 (s, 3H), 4.21–4.29 (m, 1H), 7.58–7.72 (m, 4H), 7.84 (d, 8.8 Hz, 1H), 7.89 (d, 7.2 Hz, 2H), 8.30 (s, 2H), 10.16 (s, 1H); ^{13}C NMR [100 MHz, $(CD_3)_2SO$] 18.1, 19.5, 28.1, 43.5, 43.7, 59.7, 115.6, 121.3, 123.1 (q, 272.4 Hz), 127.9, 129.2, 130.0 (q, 32.7 Hz), 132.6, 139.3, 141.8, 179.9; ^{19}F NMR [376 MHz, $(CD_3)_2SO$] −61.69 (s, 6F); IR 3307 (w), 2963 (w), 1525 (m), 1470 (m), 1382 (s), 1274 (s), 1219 (w), 1172 (s), 1124 (s), 979 (m), 883 (m), 847 (w), 788 (w), 742 (m), 681 (s); MS (EI) 512 (6) $[M + H]^+$, 492 (15), 371 (87), 354 (46), 338 (82), 223 (50), 208 (59), 168 (82), 156 (100), 141 (71), 125 (18); MS (CI) 512 (100) $[M + H]^+$; EA calcd. (for $C_{21}H_{23}F_6N_3OS_2$) C 49.31, H 4.53, N 8.21, found C 49.55, H 4.56, N 8.19.

(R_S,S_C)-*N*-[3,5-Bis(trifluoromethyl)phenyl]-*N*'-[1'-(*S*-methyl-*S*-phenylsulfonimidoyl-methyl)-3'-methylbutyl]thiourea [(R_S,S_C)-19]:

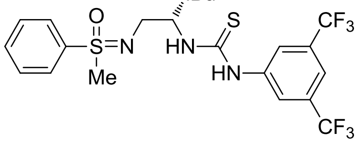

The sulfoximine (R_S,S_C)-**17** (186.3 mg, 0.5255 mmol) was dissolved in dry dichloromethane (4 mL), slowly treated with trifluoroacetic acid (1.0 mL, 13 mmol) at 0 °C, and stirred at this temperature for 2.5 h. After evaporation of all volatiles under reduced pressure the oily residue was taken up in dichloromethane and aqueous sodium hydroxide (*c* 1.25 mol/L). The

aqueous phase was extracted three times with dichloromethane, and the combined organic phases were dried over magnesium sulfate. Under reduced pressure the solvent was evaporated, and the pale yellow, oily deprotected intermediate was dried in high vacuum for 1 h. It was then dissolved in dry dichloromethane (2 mL), and 3,5-bis(trifluoromethyl)phenyl isothiocyanate (98 µL, 0.52 mmol) was dropwise syringed to this solution at 0 °C. After stirring at rt for 23 h the solvent was removed. Purification of the product by column chromatography (*n*-pentane/ethyl acetate 2/1 to 1/1 with 1% triethylamine) gave the beige solid.

Yield 87% (240.1 mg, 0.4567 mmol) over two steps; mp 57–59 °C; $[\alpha]_D$ −75.3 (*c* 1.0, $CHCl_3$); ^1H NMR [400 MHz, $(CD_3)_2SO$] 0.90 (d, 6.5 Hz, 6H), 1.51 (t, 6.9 Hz, 2H), 1.57–1.68 (m, 1H), 2.79 (dd, 11.9 Hz, 5.5 Hz, 1H), 2.98 (dd, 12.0 Hz, 3.6 Hz, 1H), 3.18 (s, 3H), 4.47 (s br, 1H), 7.54–7.67 (m, 3H), 7.71 (s, 1H), 7.88 (d, 7.3 Hz, 2H), 7.97 (d, 8.4 Hz, 1H), 8.27 (s, 2H), 10.04 (s, 1H); ^{13}C NMR [100 MHz, $(CD_3)_2SO$] 22.4, 23.0, 24.4, 40.4, 43.8, 45.9, 52.9, 115.7, 121.5, 123.1 (q, 272.2 Hz), 127.9, 128.0, 129.2, 130.0 (q, 32.5 Hz), 132.6, 139.3, 141.8, 179.3; ^{19}F NMR [376 MHz, $(CD_3)_2SO$] −61.67 (s, 6F); IR 3296 (w), 2958 (w), 1624 (w), 1528 (m), 1470 (m), 1382 (s), 1274 (s), 1173 (s), 1124 (s), 979 (m), 883 (m), 847 (w), 785 (w), 742 (m), 681 (s); MS (EI) 526 (3) $[M + H]^+$, 506 (7), 385 (56), 352 (56), 329 (12), 271 (11), 237 (34), 194 (100), 168 (79), 156 (90), 141 (65), 125 (17); MS (CI) 526 (100) $[M + H]^+$, 141 (31); EA calcd. (for $C_{22}H_{25}F_6N_3OS_2$) C 50.28, H 4.79, N 8.00, found C 50.20, H 4.64, N 7.74.

6-(Methoxycarbonyl)cyclohex-3-enecarboxylic acid (*rac*-5):

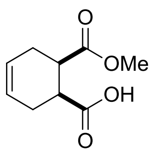

To a mixture of the cyclic *meso*-anhydride **4** (76.0 mg, 0.500 mmol) and thiourea (R_S,S_C)-**19** (13.1 mg, 0.0249 mmol) in dry methyl *tert*-butyl ether (5 mL) was added dry methanol (203 µL, 5.00 mmol). The reaction mixture was stirred at rt for 24 h. After evaporation of all volatiles under reduced pressure the oily residue was taken up in ethyl acetate and washed with aqueous hydrochloric acid (*c* 2 mol/L). The organic phase was dried over magnesium sulfate. Evaporation of the solvent under reduced pressure afforded a yellow oil which was processed by column chromatography (*n*-pentane/diethyl ether 1/1) to give the product as a white solid.

Yield 81% (74.9 mg, 0.407 mmol); mp 83–84 °C; ^1H NMR (400 MHz, $CDCl_3$) 2.27–2.45 (m, 2H), 2.50–2.68 (m, 2H), 3.03–3.11 (m, 2H), 3.70 (s, 3H), 5.64–5.72 (m, 2H); ^{13}C NMR (100 MHz, $CDCl_3$) 25.6, 25.8, 39.5, 39.6, 52.0, 125.0, 125.1, 173.5, 179.0.

The spectroscopic data are in agreement with those described in literature [6,7]. Since the product showed no optical rotation, it was converted into 3a,4,7,7a-tetrahydroisobenzofuran-1(3*H*)-one by reduction of the ester function with lithium triethylborohydride and subsequent acid-catalysed lactonization [8]. The lactone allows GC analysis with a chiral stationary phase and confirms the racemic catalysis product: t_r = 82.3 min, t_r = 83.0 min (Lipodex-E, isotherm 100 °C for 50 min, then increase of 3 °C/min, H_2), er = 50:50.

(*S*)-5-Ethoxycarbonyl-6-methyl-4-phenyl-3,4-dihydropyrimidin-2(1*H*)-one [(*S*)-20]:

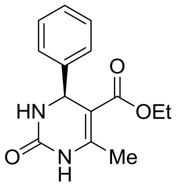

Benzaldehyde (38 μL, 0.38 mmol), urea (15.0 mg, 0.250 mmol), and trifluoroacetic acid (1.9 μL, 0.025 mmol) were dissolved in dry dichloromethane (10 mL), and the reaction mixture was stirred at rt for 1 h. Then, thiourea (*S*)-**12** (12.9 mg, 0.0249 mmol) and ethyl acetoacetate (96 μL, 0.75 mmol) were added to the solution, and stirring was continued at rt. After 5 d the solvent was evaporated under reduced pressure, and the remaining solid residue was washed with a small amount of cold ethyl acetate. The product was obtained as white solid and separated from the solvent by centrifugation (5300 rpm, 3 min).

Yield 92% (59.6 mg, 0.229 mmol); mp 202–204 °C; $[\alpha]_D$ 22.0 (*c* 0.05, iPrOH); ^{1}H NMR [400 MHz, $(CD_3)_2SO$] 1.09 (t, 7.1 Hz, 3H), 2.24 (s, 3H), 3.98 (q, 7.1 Hz, 2H), 5.14 (d, 3.3 Hz, 1H), 7.21–7.27 (m, 3H), 7.29–7.37 (m, 2H), 7.73 (s, 1H), 9.18 (s, 1H); ^{13}C NMR [100 MHz, $(CD_3)_2SO$] 14.1, 17.8, 53.9, 59.1, 99.1, 126.1, 127.1, 128.2, 144.7, 148.2, 151.9, 165.1; HPLC t_r = 9.8 min, t_r = 12.4 min (Chiralcel OD-H, 0.7 mL/min, *n*-heptane/isopropanol = 80/20, λ = 230 nm, 20 °C), er = 72:28.

The spectroscopic data match those described in literature [9,10]. The absolute configuration of the enantioenriched product was determined to be (*S*) by comparison with the reported literature value of $[\alpha]_D$ 63 (*c* 0.5, MeOH, er = 97:3) [11].

References

1. Brandt, J.; Gais, H.-J. *Tetrahedron: Asymmetry* **1997**, *6*, 909–912. doi:10.1016/S0957-4166(97)00046-3
2. Shiner, C. S.; Berks, A. H. *J. Org. Chem.* **1988**, *53*, 5542–5545. doi:10.1021/jo00258a029
3. Wade, P. A.; Le, H.; Amin, N. V. *J. Org. Chem.* **2002**, *67*, 2859–2863. doi:10.1021/jo010924k
4. Frings, M.; Atodiresei, I.; Wang, Y.; Runsink, J.; Raabe, G.; Bolm, C. *Chem. Eur. J.* **2010**, *16*, 4577–4587. doi:10.1002/chem.200903077
5. Bolm, C.; Hackenberger, C. P. R.; Simić, O.; Verrucci, M.; Müller, D.; Bienewald, F. *Synthesis* **2002**, 879–887. doi:10.1055/s-2002-28514
6. Bolm, C.; Schiffers, I.; Dinter, C. L.; Gerlach, A. *J. Org. Chem.* **2000**, *65*, 6984–6991. doi:10.1021/jo000638t
7. Campbell, C. L.; Hassler, C.; Ko, S. S.; Voss, M. E.; Guaciaro, M. A.; Carter, P. H.; Cherney, R. J. *J. Org. Chem.* **2009**, *74*, 6368–6370. doi:10.1021/jo9011249
8. Jaeschke, G.; Seebach, D. *J. Org. Chem.* **1998**, *63*, 1190–1197. doi:10.1021/jo971731t
9. Martínez, S.; Meseguer, M.; Casas, L.; Rodríguez, E.; Molins, E.; Moreno-Mañas, M.; Roig, A.; Sebastián, R. M.; Vallribera, A. *Tetrahedron* **2003**, *59*, 1553–1556. doi:10.1016/S0040-4020(03)00051-6
10. González-Olvera, R.; Demare, P.; Regla, I.; Juaristi, E. *Arkivoc* **2008**, (*vi*), 61–72.
11. Wang, Y.; Yang, H.; Yu, J.; Miao, Z.; Chen, R. *Adv. Synth. Catal.* **2009**, *351*, 3057–3062. doi:10.1002/adsc.200900597
